# Indigenous Cancer Research: Reflections on Roles and Responsibilities

**DOI:** 10.1200/JGO.19.00124

**Published:** 2020-01-13

**Authors:** Nina Scott, Hayley Bennett, Bridgette Masters-Awatere, Diana Sarfati, Polly Atatoa-Carr, Ricci Harris

**Affiliations:** ^1^ Waikato District Health Board, Hamilton, New Zealand; ^2^ Rotorua, New Zealand; ^3^ University of Waikato, Hamilton, New Zealand; ^4^ University of Otago Wellington, Wellington, New Zealand

## INTRODUCTION

When asked for a commentary on the roles and responsibilities of non-Indigenous researchers in Indigenous cancer research, several potential non-Indigenous coauthors were contacted. The majority wanted to “run for the hills,” signifying that many researchers feel uncomfortable, out of their depth, and fearful regarding methodologies and principles for safe and effective research with and for Indigenous peoples.

Most health research is relevant for Indigenous peoples, and most researchers are non-Indigenous. Thus, if researchers don’t engage with Indigenous research principles, health research can and does result in significant harm for Indigenous peoples.^1,2^ Nonengagement may also prevent potential positive outcomes for Indigenous communities (eg, health gain, workforce and community development) and impinge on Indigenous rights to health and development.^3^

In this commentary, we outline our reflections and observations on the roles and responsibilities of non-Indigenous cancer researchers and identify some of the principles that guide researchers in Aotearoa (Aotearoa is the name given by the Indigenous Māori for New Zealand). We do not attempt to provide an in-depth analysis or comprehensive best practice summary, but hope to encourage more discussion in this critical and evolving area.

## THE AOTEAROA CONTEXT

Aotearoa New Zealand is a country of just under 5 million people, with Māori (Māori are the Indigenous people of Aotearoa) comprising 15.2% of the population.^4^ European colonization led to dispossession of land and resources, Indigenous marginalization and discrimination, and the privileging of non-Indigenous ways of knowing and doing (in this commentary, we refer to non-Indigenous New Zealanders of primarily European descent as non-Māori or Pākehā; given the colonial context of Aotearoa, with power and privilege afforded to Pākehā, we are not referring to New Zealanders from the Pacific Islands, Asia, or recent migrants or refugees who are also non-Maori).^2,5,6^ This has resulted in persistent inequities in the distribution of resources and the determinants of health (including housing, employment, and income) for Māori.^5,6^ Additionally, health systems advantage non-Māori and create barriers to health care and poorer quality of care for Māori.^6-14^ The result is higher morbidity and mortality for Māori compared with non-Māori across most diseases, including cancer.^7,15-17^ Māori are more likely to be diagnosed with cancer and nearly twice as likely to die as a result of cancer.^18^ Survival inequities for Māori persist, even after adjusting for factors like age, cancer grade, stage, and comorbidity.^18,19^

There are some positive examples of equity being addressed in Aotearoa,^20^ but government agencies (and others) continue to resource programs that grow the equity gap and withhold or remove resources that could improve Māori health and development.^13,14^ Studies have shown health services to be a significant contributor to cancer inequities for Māori.^19,21-25^

## EQUITY RESPONSIBILITIES OF ALL HEALTH RESEARCHERS AND PRACTITIONERS

Te Tiriti o Waitangi (Te Tiriti; the Treaty of Waitangi) was signed in 1840 by Māori and the British Crown (now represented by the New Zealand Government).^26^ As Te Tiriti partners, the Government has obligations to ensure equitable health outcomes for Māori. Te Tiriti also supports Māori rights to self-determination. Although Te Tiriti is Aotearoa specific, other documents like the UN Declaration on the Rights of Indigenous Peoples (UNDRIP) apply to Indigenous peoples worldwide.^3^ Both the UNDRIP and Te Tiriti provide guidance on the Indigenous right to health, equity, and self-determination, as well as ”necessary steps” for states (or government) to ensure that Indigenous people realize these rights. Some of these necessary steps for researchers are outlined in this commentary.

## HEALTH RESEARCH APPROACHES IN AOTEAROA

Health research in Aotearoa encompasses a spectrum of engagement with Māori health and equity responsibilities.^27,28^ At one end is Kaupapa Māori research (KMR): it is Māori led, centers on Māori, and views Māori as the norm.^1,29-32^ The critical elements of KMR theory include an analysis of power structures, whereby colonization, racism, and the non-neutrality of systems (including health) are examined.^32^ Hei Āhuru Mōwai–National Māori Cancer Leadership Aotearoa recommend the detection, prevention, and eradication of racism within cancer control at all levels. In research, this includes recruiting staff with antiracism experience, examining research staffing and funding by ethnicity, and training staff in cultural safety. Examples of such research in Aotearoa include cancer support research within Māori health organizations^33^ and work by Māori researchers to correct the Māori undercount in national cancer statistics, providing accurate Māori cancer epidemiology.^34,35^

In the middle of the spectrum is research that incorporates a degree of equity responsiveness, where Māori and non-Māori are involved (to varying degrees) in governance and decision making, as well as in research roles. An example is the University of Otago Wellington Cancer and Chronic Conditions Research team.^36,37^

At the other end of the spectrum is research that is not designed or conducted to meet equity and Māori health responsibilities. This research includes an absent (or superficial) consideration of Māori health or even research that is overtly victim blaming^38^ and/or increases inequities.

## PRINCIPLES FOR SAFE AND EFFECTIVE RESEARCH IN THE AOTEAROA CONTEXT

Māori academics have described the importance of decolonized and Kaupapa Māori theory-informed or aligned practice for Indigenous and non-Indigenous researchers.^1^ This practice considers that research must benefit Māori, for example, through research governance, focus, methodology, Māori capacity building, and ultimately, findings, dissemination, and implementation. The research process and outcomes should be aimed at positive transformation.^1^ Smith et al^39^ have suggested that the following questions be asked:

- Who has helped define the research problem?
- For whom is this study worthy and relevant? Who says so?
- Which cultural group will be the one to gain new knowledge from this study?
- To whom is the research accountable?
- Who will gain most from the study?

If Māori are not governing research, there should be partnership agreements about decision making and what happens when there are disagreements.^40^ Discussion about recruitment of Māori participants, collection and analysis of Māori data, and interpretation of findings are essential. These partnerships require quality relationships between coinvestigators, students, advisors, partners, governors, and communities, which are maintained over time.^1,32,41^

Research should include principles of Indigenous data sovereignty,^42^ collect high-quality ethnicity data,^43^ and where possible, Māori data analysis should be to the same depth and breadth as non-Māori data analysis (eg, equal explanatory power in quantitative studies).^44^ Research methods (qualitative and/or quantitative) should enable maximum Māori health and development gain and ensure Māori perspectives and realities are heard. The interpretation should be non–victim-blaming and reject cultural deficit theories. Framing should include critical analysis of structural determinants of health, racism, privilege, and power.^1,32^

Redistribution of power from non-Indigenous to Indigenous research bodies and researchers is central to a decolonizing approach and in line with Indigenous rights to self-determination. This approach recognizes that research academies are commonly viewed as colonial structures, where non-Indigenous voices have been dominant in research and Indigenous voices have been silenced. It is important for non-Indigenous researchers to support Indigenous capacity and Indigenous research leadership, including stepping aside and handing over leadership.

Cultural safety principles apply to research organizations and practitioners. Cultural safety involves understanding one’s own culture, values, beliefs, and biases; understanding colonization, racism, white privilege, and power imbalances; and a commitment to continuous critical self-reflection and learning.^45,46^ Culturally safe organizations reflect on where power lies in the organization, analyze equity at all levels, and make resourced and monitored plans to achieve equity.^46^

Reflections from non-Māori with expert experience working in Indigenous research include these key observations^47-49^:

- Understand your position (relative power) in relation to Māori researchers and communities. Keep a mindset of humble “unknowing” rather than ”expert.”
- Be able to say who you are and where you come from and how you will determine Māori research needs, support Māori control over the research, center on Māori knowledge, and guard Māori information.
- Recognize that quality relationships are built on time and trust and require constant reflection. Relationships involve meeting obligations as well as receiving benefits.
- Honor a responsibility to Māori workforce development.
- Listen. You will be judged on what you do as well as what you say.
- Be prepared to decentralize Western European epistemology to make way for other ways of knowing and doing.
- Be prepared to guide and support, and not control.

Alongside these insights, many tools and frameworks can assist research with and for Indigenous people.^27,28,50^

## OUR CHALLENGES, SUCCESSES, AND OBSERVATIONS

There are examples of research in Aotearoa, where these principles outlined above for safe and effective research are demonstrated.^36,37,51,52^ However, a number of researchers and institutions in Aotearoa remain poorly engaged, as evidenced by research proposals reviewed by the authors over the years. Many proposals, including those in cancer research, have the potential to increase inequities. Often, the equity gap will be increased simply because the research will create more benefit for non-Indigenous than Indigenous groups.

The Health Research Council of New Zealand sets aside some funding for Māori-led research and has an expectation that all research contributes to improving Māori health. However, until the recent introduction of Maori health advancement criteria for non–Māori-led research, Māori health did not contribute to the scoring that determined whether a proposal was fundable.^53^ The overall proportion of research funding in Aotearoa put toward improving Māori cancer outcomes is difficult to estimate, but data for the last 5 years from one funder suggests less than 5%.^54^

This highlights the importance of monitoring and evaluating research institutions, proposals, funding, and practitioners to ensure principles for safe and effective research with and for Māori are enacted. All health research in Aotearoa must meet ethical standards, including reflecting on Te Tiriti principles, promoting Māori well-being, and ensuring Māori participation in research and ethical review. Work to strengthen the ethical standards to ensure greater focus on improving Māori health is ongoing.^50,55^

It is critical to consider power, privilege, and partnership in the Indigenous, non-Indigenous research space. Partnerships are good, but are not always equal; therefore, privileging the Indigenous partner is important.^56^ From our experience, it can be risky for Indigenous researchers in a non-Indigenous research team. Māori researchers may need to challenge supervisors and colleagues, for example, about racist or victim-blaming research design and interpretation. They may also experience an unfair burden of responsibility and expectation of being an expert in all things Māori or “representing” all Māori.

Resourcing quality power-sharing relationships needs to be built into timelines and budgets. Researchers must also take time to regularly critically reflect on power sharing arrangements within their own research partnerships throughout their career. This includes checking in with Indigenous partners; if the Indigenous partners think the relationship isn’t equal, it isn’t. Indigenous research mentors, advisory groups, elders, and community advisors are good safety and support mechanisms.

It is critical to consider what research questions/foci will maximize benefit for Indigenous groups, for example, determinants of health, risk/protective factors, and cancers that have high levels of inequity and/or high levels of mortality and morbidity for Indigenous peoples. If the creation of inequities is inevitable (more benefit for non-Indigenous than Indigenous) it is important to think of ways to enhance Indigenous well-being and development in other ways. For example, in the new national bowel cancer screening program in Aotearoa, equity interventions for Māori have been considered and include mechanisms for increasing Maori participation, adding health gain for Māori along the screening pathway (eg, smoking cessation), and considering lowering the eligibility age range for Māori.^57^

Gaining maximum equity and Indigenous well-being starts well before dissemination of research. An Indigenous integrated knowledge translation plan, outlined in the He Pikinga Waiora Māori Implementation Framework, encourages engagement with potential end users to codesign knowledge sharing plans at the start of the research process.^58^

In conclusion, non-Indigenous researchers who are decolonizing, focused on achieving health gain and equity for Indigenous people, and do not appropriate or dominate Indigenous research autonomy are essential. Working in this way will strengthen researchers and increase the likelihood that research will have a positive impact.

We recognize that research in Aotearoa, particularly cancer research, has gained important Indigenous responsiveness over the last 20 years, but there is still much more progress to be made. We hope this commentary promotes additional discussion, debate, and impetus for researchers in Aotearoa and worldwide to contribute to the realization of the right to the highest attainable standard of health^3^ for Indigenous peoples.
